# Chemical Constituents and Biological Potentials of *Teucrium scordium* subsp. *scordioides* Extracts: In Vitro Experimental and In Silico Perspectives

**DOI:** 10.1002/open.70216

**Published:** 2026-04-28

**Authors:** Bengusu Hacer Akgul, Sakina Yagi, Mehmet Veysi Cetiz, Enver Saka, Gokhan Zengin, Eulogio J. Llorent‐Martínez, Marina Gómez‐Torres, Ismail Yapıcı, Ilhami Gulcin, Evren Yıldıztugay

**Affiliations:** ^1^ Department of Biology Science Faculty Selcuk University Konya Turkey; ^2^ Department of Botany Faculty of Science University of Khartoum Khartoum Sudan; ^3^ Department of Medical Biochemistry Faculty of Medicine Harran University Sanliurfa Turkey; ^4^ Department of Physical and Analytical Chemistry Faculty of Experimental Sciences University of Jaén Jaén Spain; ^5^ Gümüşhane University Central Research Laboratory Gümüşhane Türkiye; ^6^ Department of Chemistry, Science Faculty Ataturk University Erzurum Turkey; ^7^ Rectorate of Agri İbrahim Çeçen University Agri Türkiye; ^8^ Department of Biotechnology, Science Faculty Selcuk University Konya Turkey

**Keywords:** antioxidant, chemical profile, enzyme inhibition, molecular docking, molecular dynamics simulations, *Teucrium scordium*

## Abstract

This study was undertaken to examine, for the first time, the antioxidant and enzyme inhibitory properties of several extracts obtained from the aerial parts of *Teucrium scordium* subsp. *scordioides* (Schreb.) Arcang (family: Lamiaceae). Among the extracts, the EtOH extract contained the highest total phenolic (89.96 mg  GAE/g) and flavonoid (45.74 mg  RE/g) contents. Phenylethanoid glycosides were predominant, particularly in the ethanol and aqueous extracts, and teucroside/forsythoside B and verbascoside/forsythoside A were tentatively identified as the major compounds. EtOH and 70% EtOH extracts showed comparable (*p* > 0.05) anti‐DPPH activity (201.89 and 188.52 mg  TE/g), Cu^++^ (562.81 and 547.88 mg  TE/g), and Fe^+++^ (329.13 and 320.08 mg  TE/g) reducing capacities. The EtOH extract showed the best antityrosinase activity (72.00 mg   KAE/g). The EtOAc extract inhibited human carbonic anhydrase (CA) isoenzymes I and CAII (IC_50_ = 26.86 and 65.37 μg/mL, respectively). Complementary in silico analyses of the major phenylethanoid and flavonoid glycosides supported their possible multitarget interaction patterns and identified the Forsythoside A–BChE complex as a computationally favorable model for future experimental validation. Overall, these findings indicate that *T. scordium* subsp. *scordioides* may represent a natural source of antioxidants and enzyme inhibitors for use in different health‐promoting formulations.

## Graphical abstract

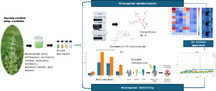

## 1 Introduction

*Teucrium L.* (Lamiaceae) is a species‐rich genus comprising ≈300  taxa, classified into eight subsections and occurring across Europe, North Africa, and temperate Asia*.* The Mediterranean basin is widely recognized as a major center of *Teucrium* species diversity [1]. Most *Teucrium* species are perennial shrubs with creeping rootstocks, typically inhabiting rocky or dry, stony habitats. Traditionally, they are used to treat cold, fever, digestive problems, cardiac diseases, diabetes, wound healing, genitourinary, and neurological disorders [2]. Pharmacologically, many *Teucrium* species possess anticancer, antioxidant, antidiabetic, antimicrobial, anti‐inflammatory, and insecticidal activities. Phytochemical investigations across the genus have reported terpenoids, steroids, flavonoids, iridoids, clerodanes, and phenylethanoid glycosides as recurrent metabolite classes. However, for many taxa, integrated profiling that links extract chemistry with multienzyme inhibition and antioxidant endpoints remains limited, warranting a systematic evaluation [3, 4, 5].

In Turkey, the genus *Teucrium* is represented by ≈34 species (46  taxa), with endemics accounting for ≈35% [6, 7]. *T. scordium* subsp*. scordioides* (*Schreb*.) Arcang., commonly known as water germander, is a perennial herb bearing tubular lilac‐to‐purple flowers arranged in clusters*.* Traditionally, it is used to treat wounds, fever, respiratory problems, and intestinal parasites [2]. From the aerial parts, several flavonoids (cirsiliol, cirsimaritin, cirsilineol, and luteolin) and diterpenes (6‐hydroxyteuscordin, 2,6‐dihydroxyteuscordin, 2‐keto‐19‐hydroxyteuscordin and teuscordin) have been reported [8, 9, 10, 11]. The essential oil of the aerial parts is rich in germacrene D, *d*‐cadinene, alloaromadendrene, caryophyllene oxide, *α*‐pinene, and *β*‐pinene, and has been associated with antifungal, anti‐inflammatory, and anti‐migratory effects [5, 12, 13, 14, 15, 16, 17]. Cyclohexane, dichloromethane, and methanol extracts of the aerial parts have shown notable anticancer and antimicrobial activities [18]. More recently, neo‐clerodane diterpenoids (scordidesin A, teucrin A, and 6‐ketoteuscordin) were isolated from the aerial parts and displayed moderate antimicrobial activity [19]. In the present work, we evaluated the antioxidant capacity and enzyme inhibitory potential of different extracts obtained from the aerial parts of *T. scordium* subsp. *scordioides*. In addition, an in silico workflow was applied, combining molecular docking, 100 ns molecular dynamics (MD) simulations, and MM/GBSA analysis of the major phenylethanoid and flavonoid glycosides. This strategy was used to explore the putative multitarget interaction profiles of the major glycosides at the molecular level. Antioxidant activity was assessed through free‐radical scavenging, metal‐chelation, and metal‐ion reducing assays, whereas enzyme inhibition was evaluated against acetylcholinesterase (AChE), butyrylcholinesterase (BChE), tyrosinase, *α*‐amylase and *α*‐glucosidase, as well as human carbonic anhydrase (CA) isoenzymes I and II. se and *α*‐glucosidase enzymes as well as the human carbonic anhydrase isoenzymes I and II. Also, a comprehensive investigation on the chemical profile of different extracts was performed by HPLC–ESI–Q–TOF‐MS.

## 2 Materials and Methods

### 2.1 Plant Collection

Plant specimens were collected in 2022 from the Ulumuhsine area (Konya, Turkey, GPS coordinates: 37°55′25.78″N and 32°14′58.76″E) at an elevation of 1150 m. Dr Evren Yildiztugay carried out the formal botanical classification of the material. A voucher specimen, under the code EY‐3187, was placed in the Faculty of Science (in KNYA herbarium) at Selçuk University. After collection, the aerial parts were separated immediately and air‐dried at room temperature in a shaded, well‐ventilated area. After complete drying, the plant material was milled to a fine powder. To ensure chemical integrity and prevent deterioration, the powdered samples were sealed in opaque containers and stored under stable conditions.

### 2.2 Plant Extract Preparation

Four solvent systems were used to obtain bioactive constituents: ethyl acetate, ethanol, 70% (v/v) ethanol–water, and distilled water. For each extraction, 10 g of the prepared plant material was mixed with 200 mL of the corresponding solvent. The methodology was adapted according to the properties of the solvent. Extractions using organic solvents were performed via maceration for 24 h under ambient conditions. In contrast, the water‐based extraction utilized a 15‐min infusion with heated water. Following extraction, the products obtained were concentrated using distinct techniques. The aqueous extract was stabilized by lyophilization, while the organic solvent fractions were recovered by evaporating the solvents under reduced pressure using a rotary evaporator.

### 2.3 Total Phenolic and Flavonoid Contents

Total phenolic and flavonoid contents of the extracts were determined using established colorimetric assays, following the referenced procedure. Calibration curves were constructed using gallic acid (mg gallic acid equivalents (GAE)/g) and rutin (mg rutin equivalents (RE)/g) as standards [20].

### 2.4 Phytochemical Profiling by HPLC–ESI–Q–TOF–MS

Approximately 5 mg of each dried extract was dissolved in 1 mL methanol; the aqueous extract was reconstituted in 1 mL of 10% MeOH. All solutions were passed through 0.45 μm membrane filters. Analyses were performed on an Agilent 1200 system (Agilent Technologies, Santa Clara, CA, USA) coupled to an Agilent 6530B Q–TOF–MS for compound characterization [21, 22]. Full analytical parameters are provided in the Supporting Information.

### 2.5 Antioxidant Assays

Antioxidant capacity was assessed using a panel of in vitro assays, following a previously described protocol [23]. Reducing power was evaluated using FRAP (ferric reducing antioxidant power) and CUPRAC (cupric reducing antioxidant capacity), while radical scavenging was measured using DPPH (2,2‐diphenyl‐1‐picrylhydrazyl) and ABTS (2,2′‐azino‐bis(3‐ethylbenzothiazoline‐6‐sulfonic acid)) assays. Results from these four assays were quantified and standardized to Trolox and reported as mg Trolox equivalents (TE) per g of dried extract (mg  TE/g). Trolox and expressed as milligrams of Trolox equivalent per gram of dried extract (mg  TE/g). Total antioxidant capacity was additionally determined by the phosphomolybdenum method (PBD assay) and expressed as mmol Trolox equivalents per g (mmol  TE/g). Finally, metal‐chelating activity was evaluated using a chelation assay and reported as mg EDTA equivalents (EDTAE) per g of extract (mg EDTAE/g).

### 2.6 Enzyme Inhibitory Activity Assays

The enzyme inhibitory potential of the extracts was evaluated against five key targets; acetylcholinesterase (AChE), butyrylcholinesterase (BChE), tyrosinase, *α*‐amylase and *α*‐glucosidase; using established colorimetric procedures [23]. To standardize the results, inhibition was quantified using established reference compounds. Cholinesterase inhibitory activity was expressed as mg galanthamine equivalents (GALAE) per g of extract (mg  GALAE/g). *α*‐Amylase and *α*‐glucosidase inhibitory activities were expressed as mg acarbose equivalents (ACAE) per g (mg  ACAE/g), while tyrosinase inhibition was reported as mg Kojic acid equivalents (KAE) per g (mg  KAE/g). In addition, the extracts were evaluated for inhibitory activity against human carbonic anhydrase isoenzymes I and II (hCA I and hCA II).

### 2.7 Molecular Docking

Molecular docking was performed to assess the binding tendencies of the phenylethanoid and flavonoid glycosides identified in *Teucrium scordium* subsp. *scordioides* (Schreb.) Arcang. Docking calculations were conducted for seven targets: AChE, BChE, *α*‐amylase, *α*‐glucosidase, hCA I, hCA II, and tyrosinase. The three‐dimensional crystal structures of the enzymes were obtained from the Protein Data Bank (PDB). Ligand structures were drawn in ChemDraw and energy minimized in Avogadro v1.2.0 using the MMFF94 force field. Protein preparation was carried out using BIOVIA Discovery Studio and AutoDockTools v4.2.6. Ligand geometries were optimized in Avogadro v1.2.0; nonpolar hydrogens were merged and Gasteiger partial charges were assigned before docking. Docking runs were performed in AutoDock Vina v1.1.2 with the exhaustiveness parameter set to 32. The grid box parameters used for docking were as follows: BChE (PDB ID: 6EQP; center: 32.16, −16.33, 40.73; grid size: 25 × 25 × 25), amylase (PDB ID: 2QV4; center: 14.188, 48.964, 22.886; grid size: 28 × 28 × 24), glucosidase (PDB ID: 7KBJ; center: −18.79, 2.18, 15.75; grid size: 25 × 25 × 25), hCA I (PDB ID: 3LXE; center: −19.049, 36.885, 44.812; grid size: 40 × 40 × 40), tyrosinase (PDB ID: 6QXD; center: 21.81, 12.22, 91.40; grid size: 25 × 25 × 25), AChE (PDB ID: 7E3H; center: −54.44, 32.90, −28.65; grid size: 25 × 25 × 25), and hCA II (PDB ID: 4IWZ; center: 14.059, 4.757, 14.338; grid size: 40 × 40 × 40). Binding pockets were defined using cavity predictions from POCASA v1.1. For protocol validation, cocrystallized ligands were redocked into their native binding sites, and root mean square deviation (RMSD) values were calculated to assess pose reproducibility [24, 25]. Protein–ligand contact patterns, including hydrogen bonds, hydrophobic contacts, and *π*‐stacking interactions, were analyzed using the protein–ligand interaction profiler [26, 27].

### 2.8 Molecular Dynamics Simulation

MD simulations were conducted to probe the dynamic stability and binding behavior of selected protein–ligand complexes. Initial system setup was generated using CHARMM‐GUI (https://charmm‐gui.org) [28, 29]. Proteins and ligands were parameterized with the CHARMM36m force field [30], solvated in an explicit TIP3P (three‐site transferable intermolecular potential) water box, and neutralized by adding counterions. The ionic strength was maintained at 0.15 M NaCl. Nonbonded interactions were treated using the Verlet cutoff scheme, and bonds involving hydrogen atoms were constrained using linear constraint solver. Long‐range electrostatics were computed using the particle mesh Ewald method. Energy minimization was performed using the steepest–descent algorithm until the maximum force dropped below 1000 kJ/mol/nm. Equilibration was carried out in two stages: first under NVT (constant number of particles, volume, and temperature) and then under NPT (constant number of particles, pressure, and temperature) at 310 K. Production runs were performed for 100 ns using GROMACS v2024.3. After the simulations, binding‐free energies were estimated using gmx_MMPBSA to provide comparative estimates of ligand affinity and complex stability [31, 32].

### 2.9 Statistical Analysis

All experiments were performed in triplicate (*n* = 3, as technical replications), and results are expressed as mean  ±  standard deviation (SD). Statistical differences among extracts were evaluated using one‐way analysis of variance (ANOVA) in GraphPad Prism version 9.2, followed by Tukey's post hoc test for multiple comparisons. Exact *p*‐values are provided where applicable, and statistically significant differences between groups are indicated using different superscript letters. A value of *p * <  0.05 was considered statistically significant.

## 3 Results and Discussion

### 3.1 Total Phenolic and Total Flavonoid Contents

Total phenolic content (TPC) and total flavonoid content (TFC) were determined for the different extracts of *T. scordium* subsp. *scordioides*, and the results are shown in Table 1. TPC values ranged from 32.94 to 89.96 mg  GAE/g, following the order EtOH > 70% EtOH  >  H_2_O  >  EtOAc. TFC values ranged from 10.50 to 45.74 mg  RE/g and ranked as EtOH > 70% EtOH  ≈  EtOAc  >  H_2_O. Accordingly, EtOH was the most effective solvent for obtaining the highest TPC and TFC levels. The TPC and TFC of *T. scordium* subsp. *scordioides* grown in Serbia were previously determined and authors reported that methanol and acetone yielded the highest TPC (157.89 mg  GAE/g) and TFC (111.93 mg rutin E/g), respectively. Differences between those values and the present results may be attributed to genetic and environmental factors, as well as the solvent used for extraction [33].

**TABLE 1:** Total phenolic and flavonoid contents in extracts of *Teucrium scordium* subsp. *scordioides* aerial parts.

Extracts	TPC, mg GAE/g	TFC, mg RE/g
EtOAc	32.94 ± 0.24^d^	24.31 ± 0.53^b^
EtOH	89.96 ± 1.39^a^	45.74 ± 0.42^a^
70% EtOH	83.46 ± 0.45^b^	23.41 ± 0.42^b^
Water	60.42 ± 0.46^c^	10.50 ± 0.30^c^

*Note:* Values are reported as mean ± SD of three parallel measurements. Different letters indicate significant differences between the tested extracts (“a” indicates the highest content, *p* < 0.05).

Abbreviations: GAE, gallic acid equivalents; RE, rutin equivalents.

### 3.2 Phytochemical Profile by HPLC–ESI–Q–TOF–MS

Phenolic compounds were identified by integrating data from commercial reference standards, high‐resolution mass spectrometry, and a comprehensive metabolite database. The reference standards, which included citric, chlorogenic, neochlorogenic, coumaric, and syringic acids, as well as luteolin, quercetin, and isorhamnetin, were analyzed individually. Their chromatographic retention times, exact masses, and tandem mass spectrometry (MS/MS) fragmentation patterns at multiple collision energies (10, 20, and 40 V) were used to identify the corresponding compounds in the plant extracts.

For compounds lacking direct standards, such as glycosylated flavonoids, identification relied on high‐resolution accurate mass and MS/MS fragmentation. Confirmation was achieved by analyzing the characteristic fragmentation of known aglycone cores, while observed neutral losses indicated the nature and linkage of attached sugar moieties. All matches were verified against spectral databases and relevant scientific literature.

The key annotations (retention times, observed deprotonated molecular ions ([M–H]^−^), proposed molecular formulas, mass accuracy (ppm error), and diagnostic fragment ions) are shown in Table 2. Brief notes on the identified compounds are provided below. Compound 1 showed a deprotonated molecular ion at *m*/*z* 341, indicative of a dihexoside (likely a diglucoside), and its fragmentation pattern supported the presence of hexoside moieties. Since HCl adducts were observed in all extracts, they were considered in the relative quantification. Several organic acids, including phenolic acids, were identified in the analyzed extracts. Compounds 2 and 4 displayed the same [M–H]^−^ ions and closely similar fragmentation patterns, consistent with (iso)citric acid. A citric acid analytical standard was used to distinguish the isomers based on retention time. Compound 3 was characterized as malic acid based on its molecular formula and a base peak at *m*/*z* 115. Compound 7 showed a neutral loss of 162 Da (hexoside), yielding an ion at *m*/*z* 153 (base peak at *m*/*z* 109); it was hence characterized as dihydroxybenzoic acid‐*O*‐hexoside. In the same way, compound 9 exhibited the deprotonated molecular ion at *m*/*z* 153, which corresponded to dihydroxybenzoic acid (its fragmentation was compared with an analytical standard of protocatechuic acid). Three caffeoylquinic acids (compounds **6**, **11**, and **13**) were characterized based on the [M–H]^−^ at *m/z* 353 and fragment ion at *m/z* 191. Neochlorogenic and chlorogenic acids were unambiguously identified by comparison with their analytical standards. Compound 28 showed a deprotonated molecular ion at *m*/*z* 515 and a base peak at *m*/*z* 353, supporting its characterization as a dicaffeoylquinic acid. Compounds 8 and 10 exhibited deprotonated molecular ions at *m*/*z* 359 and 325, respectively, and showed a neutral loss of 162 Da, yielding fragments consistent with syringic acid (*m*/*z* 197) and coumaric acid (*m*/*z* 163); both phenolic acids were identified using analytical standards.

**TABLE 2:** Characterization of the compounds found in the analyzed extracts of *Teucrium scardium* subsp. *scordioides* aerial parts.

No.	t_*R*_, min	Observed [M–H]^−^	Molecular formula	Error, ppm	Fragment ions	Assigned identification	EtOH	EtOH:H_2_O	H_2_O	EtAc
1	1.708	341.109	C_12_H_22_O_11_	−0.19	179.0554, 161.0448, 143.0340, 119.0342, 113.0249, 101.0241, 89.0243	Disaccharide	✓	✓	✓	✓
2	1.9	191.0196	C_6_H_8_O_7_	0.82	173.0093, 129.0203, 111.0083, 87.0090	Isocitric acid	✓	✓	✓	✓
3	2.1	133.0142	C_4_H_6_O_5_	0.13	115.0031	Malic acid	✓	✓	✓	—
4	2.58	191.0198	C_6_H_8_O_7_	−0.35	173.0095, 129.0185, 111.0084, 87.0088	Citric acid	✓	✓	✓	✓
5	3.5	315.0722	C_13_H_16_O_9_	−0.45	153.0544, 109.0285	Dihydroxybenzoic acid‐glucoside	✓	✓	✓	—
6	5.1	353.0877	C_16_H_18_O_9_	−0.09	191.0560, 179.0343, 173.0457, 135.0452	Neochlorogenic acid	✓	✓	✓	—
7	5.3	153.0193	C_7_H_6_O_4_	0.11	109.0292	Dihydroxybenzoic acid	✓	✓	✓	✓
8	5.8	359.0985	C_15_H_20_O_10_	0.39	197.0430	Syringic acid‐*O*‐glucoside	✓	✓	✓	✓
9	7.1	137.0242	C_7_H_6_O_3_	1.42	109.0246, 92.0273	Hydroxybenzoic acid	✓	✓	✓	✓
10	7.7	325.0924	C_15_H_18_O_8_	1.2	163.0393, 119.0500	Coumaric acid‐*O*‐glucoside	✓	✓	✓	—
11	8.8	353.0877	C_16_H_18_O_9_	0.3	191.0556, 179.0344, 173.0447	Chlorogenic acid	✓	✓	✓	—
12	9.0	405.1396	C_17_H_26_O_11_	2.54	345.1196, 179.0579, 165.0553	Unknown	✓	✓	✓	✓
13	9.3	353.0879	C_16_H_18_O_9_	0.15	191.0556	Caffeoylquinic acid	✓	✓	✓	—
14	10.7	389.1456	C_17_H_26_O_10_	0.95	331.1488, 227.0927, 167.0702	Unknown	✓	✓	✓	—
15	16.7	755.2401	C_34_H_44_O_19_	0.27	623.1969, 593.2072, 461.1656, 315.1071, 161.0240, 135.0442	Teucroside/forsythoside B	✓	✓	—	—
16	19.4	755.2402	C_34_H_44_O_19_	0.36	623.1952, 593.2078, 461.1654, 315.1073, 161.0240, 135.0448	Teucroside/forsythoside B	✓	✓	✓	✓
17	20.1	623.1979	C_29_H_36_O_15_	0.48	461.1657, 315.1086, 179.0346, 161.0239, 135.0446	Verbascoside/forsythoside A	✓	✓	✓	✓
18	20.6	785.214	C_34_H_42_O_21_	0.69	623.1968, 461.1664, 315.1064	Isorhamnetin‐*O*‐hexoside‐*O*‐rutinoside	—	✓	✓	—
19	20.7	593.1505	C_27_H_30_O_15_	1.03	447.0881, 285.0404, 243.0304, 241.0517	Luteolin‐*O*‐rutinoside	✓	✓	✓	✓
20	21.3	755.2402	C_34_H_44_O_19_	0.36	623.1958, 593.2068, 461.1650, 315.1076, 161.0239, 135.0447	Teucroside/forsythoside B	✓	✓	✓	✓
21	21.4	785.214	C_34_H_42_O_21_	0.95	623.1933, 461.1639, 315.1156	Isorhamnetin‐*O*‐hexoside‐*O*‐rutinoside	—	✓	✓	—
22	21.7	447.0923	C_21_H_20_O_11_	0.74	285.0388	Luteolin‐*O*‐hexoside	✓	✓	✓	✓
23	21.7	477.0683	C_27_H_18_O_13_	−2.13	301.0337, 178.9999, 151.0030	Quercetin‐*O*‐glucuronide	—	—	✓	—
24	22.3	623.1978	C_29_H_36_O_15_	0.59	461.1670, 315.1080, 179.0350, 161.0247, 135.0448	Verbascoside/forsythoside A	✓	✓	✓	✓
25	23.3	769.2551	C_35_H_46_O_19_	0.96	593.2075, 461.1679, 175.0407, 161.0238, 135.0448	Alysonoside	✓	✓	—	—
26	23.3	735.2139	C_34_H_40_O_18_	0.26	623.1971, 461.1657, 315.1063, 161.0266	Phenylpropanoid glycoside	✓	✓	—	✓
27	24.0	769.2541	C_35_H_46_O_19_	2.52	593.2076, 461.1646, 179.0364, 175.0393, 161.0243, 135.0440, 113.0237, 89.0242	Poliumoside	✓	✓	✓	✓
28	24.1	515.1189	C_25_H_24_O_12_	1.35	353.0872, 191.0543, 179.0342	Dicaffeoylquinic acid	✓	✓	✓	—
29	24.2	577.1558	C_27_H_30_O_14_	1.11	269.0448	Apigenin‐*O*‐rutinoside	✓	✓	—	—
30	24.3	637.2132	C_30_H_38_O_15_	0.94	461.1652, 315.1068, 161.0243, 175.0395, 135.0443	Leucosceptoside A	✓	✓	✓	✓
31	25.5	377.1603	C_20_H_26_O_7_	0.51	329.1336, 285.1516, 267.1375	Unknown	✓	✓	✓	—
32	25.6	607.1661	C_28_H_32_O_15_	1.06	299.0553, 284.0311	Diosmetin‐*O*‐rutinoside	✓	✓	—	✓
33	25.9	359.0771	C_18_H_16_O_8_	0.58	197.0448, 161.0238, 135.0454	Rosmarinic acid	✓	✓	✓	—

Phenylethanoid glycosides were the most abundant constituents. Compounds 15, 16, and 20 shared the same molecular formula and displayed similar fragmentation patterns. The MS data are consistent with both forsythoside B and teucroside, compounds that have been previously reported in different *Teucrium* species [34, 35, 36, 37]. Similarly, compounds **17** and **24** could also correspond to verbascoside or forsythoside A [34, 37]. All these compounds are phenylethanoid glycosides that share a similar core but differ in the glycosidic and esterification patterns. Compounds **25** and **27** had the same molecular formula and similar fragment ions; however, they were tentatively characterized as allysonoside and poliumoside, previously reported in *Teucrium* plants [37]; the distinction was made because allysonoside has been reported to present the base peak at *m/z* 175 [38]. Compound **26** was tentatively characterized as a phenylethanoid glycoside due to the fragmentation pattern similar to the previously discussed compounds. Compound **30** was also characterized based on previous information in *Teucrium* species [37].

Several flavonoid glycosides were characterized. In all cases, the characterization was performed based on the neutral losses of the attached moieties (162 Da for hexoside, 176 Da for glucuronide and 308 Da for rutinoside) and the aglycones at *m/z* 315, 285, 301, 269, and 299 for isorhamnetin, luteolin, quercetin, apigenin, and diosmetin, respectively.

Compound **33**, with [M–H]^−^ at *m/z* 359 and base peak at *m/z* 197, was characterized as rosmarinic acid [39].

**FIGURE 1:**
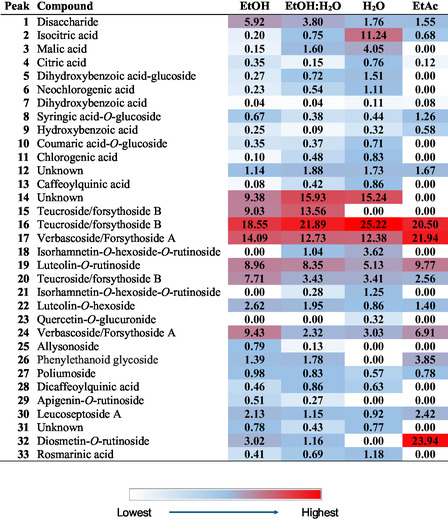
Heat map obtained by HPLC–ESI–Q–TOF of the analyzed extracts of *Teucrium scordium* subsp. *scordioides* aerial parts.

After phytochemical identification, a relative quantification was carried out for the major compounds. A heat map was generated (Figure 1) using extracted ion chromatograms (EICs) for each compound at its corresponding deprotonated molecular ion; a symmetric mass window of ±5 ppm was used for each EIC. The relative peak areas are shown in Figure 1 as percentages after normalization to the total area of all compounds within each extract.

The sum of all the phenylethanoid glycosides ranged between 45.5 % in the aqueous extract and 64% in the ethanolic extract, thus being the main chemical family of compounds in all the extracts. Among phenylethanoid compounds, teucroside/forsythoside B and verbascoside/forsythoside A were the most abundant, representing 22%–40% and 15%–29% of the total compounds. Flavonoid glycosides accounted for ≈11%–15% of all compounds, except in the ethyl acetate extract, in which the presence of compound **32** was higher. However, the highest percentage of this compound in the EA extract is due to the low extraction of the other compounds. Finally, although several phenolic acids were characterized, their contribution to the total extract was only 2%–7% of all the compounds. To sum up, the bioactivity of the extract should be mainly due to the presence of abundant phenylethanoid glycosides and, partially, to flavonoid glycosides.

### 3.3 Antioxidant Activity

In the current study, the antioxidant activity of different extracts from *T. scordium* subsp. *scordioides* aerial parts were evaluated by the DPPH, ABTS, CUPRAC, FRAP, MCA, and PBD assays. Results are shown in Table 3. Both the EtOH and 70% EtOH extracts exerted comparable (*p *≥ 0.05) anti‐DPPH (201.89 and 188.52 mg  TE/g), Cu^++^ (562.81 and 547.88 mg  TE/g), and Fe^+++^ (329.13 and 320.08 mg  TE/g) ions reducing capacity as well as total antioxidant activity (2.31 and 2.24 mmol  TE/g). The former extract displayed also the highest ABTS scavenging activity followed by the latter extract (364.61 and 287.67 mg  TE/g, respectively, *p * <  0.05). It was also noted that the ability of the EtOH and 70% EtOH extracts to scavenge radicals and reduce ions were stronger than the EtOAc extract. This observation correlates well with the TPC where both extracts had higher content than the EtOAc one. On the other hand, the aqueous extract (20.69 mg EDTAE/g) followed by the EtOAc extract (16.53 mg  EDTAE/g) exerted significant (*p * <  0.05) higher chelating power than the EtOH and 70% EtOH extracts (3.61 and 8.24 mg  EDTAE/g, respectively). No previous reports evaluating the antioxidant activity of *T. scordium* subsp. *scordioides* were found; however, other species like *T. cavernarum* displayed an anti‐DPPH activity with IC_50_ values 0.12 and 0.18 mg/mL [34]. Also, the antioxidant activity of *T. polium* extract and isolated phenolic compounds and phenylethanoid glycosides was determined using the DPPH assay and the following values were recorded; EC_50_ for butanol = 7.8  µg/mL, poliumoside B = 4.8 M, poliumoside = 7.0 M, luteolin7‐*O*‐glucoside = 13.4 lM, luteolin7‐*O*‐rutinoside = 23.7 M and teucardoside = 23.3 M [40]. However, luteolin7‐*O*‐rutinoside, teucardoside, and poliumoside were tentatively identified in the present study with the former compound accumulated in high content. Thus, it could be speculated that these compounds besides others like verbascoside, forsythoside A, forsythoside B, caffeoylquinic acid, chlorogenic acid, and hydroxybenzoic acid could collectively be responsible of the high antioxidant activity of *T. scordium* subsp. *scordioides* [41, 42, 43, 44]. Finally, the significant antioxidant activity recorded from different assays suggested that *T. scordium* subsp. *scordioides* could be an important source of natural antioxidant compounds.

**TABLE 3:** Antioxidant properties of extracts of of *Teucrium scordium* subsp. *scordioides* aerial parts.

Extracts	DPPH, mg TE/g	ABTS, mg TE/g	CUPRAC, mg TE/g	FRAP, mg TE/g	MCA, mg EDTAE/g	PBD, mmol TE/g
EtOAc	36.46 ± 0.80^b^	56.27 ± 1.45^d^	143.68 ± 1.84^c^	70.01 ± 0.60^c^	16.53 ± 1.08^b^	1.92 ± 0.17^b^
EtOH	201.89 ± 7.15^a^	364.61 ± 6.20^a^	562.81 ± 14.67^a^	329.13 ± 10.04^a^	3.61 ± 0.62^d^	2.31 ± 0.14^a^
70% EtOH	188.52 ± 18.76^a^	287.67 ± 9.30^b^	547.88 ± 4.47^a^	320.08 ± 5.17^a^	8.24 ± 0.33^c^	2.24 ± 0.09^a^
Water	60.95 ± 7.72^b^	163.59 ± 13.58^c^	286.77 ± 11.15^b^	200.86 ± 3.70^b^	20.69 ± 0.20^a^	1.58 ± 0.05^c^

*Note:* Values are reported as mean ± SD of three parallel measurements. Different letters indicate significant differences between the tested extracts ( “a” indicates the strongest ability, *p * <  0.05).

Abbreviations: EDTAE, EDTA equivalent; MCA, metal chelating activity; PBD, phosphomolybdenum; TE, Trolox equivalent.

### 3.4 Enzyme Inhibitory Activity

Enzyme inhibitors are widely used in the management of metabolic disorders and related conditions, including diabetes, cancer, obesity, osteoporosis, Alzheimer's disease, and other neurological diseases [45]. In this study, the enzyme inhibitory potential of different extracts of *T. scordium* subsp. *scordioides* was assessed against acetylcholinesterase (AChE), butyrylcholinesterase (BChE), tyrosinase (Tyr), *α*‐amylase, and *α*‐glucosidase, as well as human erythrocyte carbonic anhydrase (CA) I and CA II isoenzymes. The results are shown in Tables 4 and 5. The three organic extracts showed comparable anti‐AChE activity (2.69–2.87 mg GALAE/g, *p *≥ 0.05). In contrast, the strongest anti‐BChE activity was observed for the EtOAc extract (2.84 mg  GALAE/g), followed by the other organic extracts (2.06 and 1.78 mg  GALAE/g; *p*≥0.05). All three organic extracts also exerted notable tyrosinase inhibition (58.87–72.00 mg  KAE/g, *p* < 0.05), ranking as EtOH > 70% EtOH  >  EtOAc. Regarding diabetes‐related enzymes, all extracts inhibited *α*‐glucosidase more strongly than *α*‐amylase, with the highest activity recorded for the 70% EtOH extract (2.21 mmol  ACAE/g), followed by the EtOH extract (2.13 mmol ACAE/g), while the best *α*‐amylase inhibitory activity was obtained from the EtOAc extract (0.46 mmol  ACAE/g). The four extracts moderately inhibited the CAI and CAII isoenzymes with the best effect exerted by the EtOAc extract (IC_50_ = 26.86 and 65.37  µg/mL toward CAI and CAII, respectively).

**TABLE 4:** Enzyme inhibitory effects of of *Teucrium scordium* subsp. *scordioides* aerial parts.

Extracts	AChE, mg GALAE/g	BChE, mg GALAE/g	Tyrosinase, mg KAE/g	Amylase, mmol ACAE/g	Glucosidase, mmol ACAE/g
EtOAc	2.69 ± 0.15^a^	2.84 ± 0.46^a^	58.87 ± 1.02^c^	0.46 ± 0.01^a^	0.42 ± 0.01^d^
EtOH	2.87 ± 0.03^a^	2.06 ± 0.08^b^	72.00 ± 0.72^a^	0.34 ± 0.01^b^	2.13 ± 0.02^b^
70% EtOH	2.87 ± 0.01^a^	1.78 ± 0.03^b^	61.91 ± 0.10^b^	0.28 ± 0.01^c^	2.21 ± 0.01^a^
Water	1.16 ± 0.02^b^	0.86 ± 0.09^c^	19.30 ± 1.23^c^	0.05 ± 0.01^d^	0.48 ± 0.01^c^

*Note:* Values are reported as mean ± SD of three parallel measurements. Different letters indicate significant differences between the tested extracts (“a” indicates the strongest ability, *p * <  0.05).

Abbreviations: ACAE, acarbose equivalent; GALAE, galantamine equivalent; KAE, Kojic acid equivalent; na: not active.

**TABLE 5:** Inhibitory effects on human carbonic anhydrase isoenzymes (CA) I and CA II).

	CA I		CA II	
	IC_50_, µg/mL	*R* ^2^	IC_50_, µg/mL	*R* ^2^
EtOAc	26.86 ± 0,56^b^	0.9790	65.37 ± 4.92^b^	0.9248
EtOH	34.47 ± 0.74^c^	0.9785	81.52 ± 2.35^d^	0.9716
70% EtOH	77.00 ± 1.13^d^	0.9853	70.00 ± 4.47^c^	0.9361
Water	94.93 ± 8.24^e^	0.9132	135.88 ± 9.65^e^	0.9290
Acetazolamide (Standard inhibitor) (ng/mL)	4.23 ± 0.06^a^	0.9836	4.81 ± 0.03^a^	0.9940

*Note:* Values are reported as mean ± SD of three parallel measurements. Different letters indicate significant differences between the tested extracts (“a” indicates the strongest ability, *p * <  0.05).

The cholinesterase inhibition activity exerted by different extracts could be partly attributed to phenylethanoid glycosides. A previous study demonstrated that an extract rich in phenylethanoid glycosides, at concentration 100  µg/mL, displayed significant anti‐AChE (48.87%) and anti‐BChE (58.81%) activities [42]. Furthermore, the pure compounds forsythoside B and verbascoside were proven to be effective cholinesterases inhibitors [42, 46]. Other compounds identified in the present study like hydroxybenzoic acid [47] and rosmarinic acid [48] were also known to inhibit cholinesterase enzymes. Dicaffeoylquinic acid was shown to exert significant anti‐Tyr activity [49], while chlorogenic acid effectively inhibited both the *α*‐amylase and *α*‐glucosidase [50]. Thus, the plant could be a promising source of natural enzyme inhibitors.

### 3.5 Molecular Docking

**FIGURE 2:**
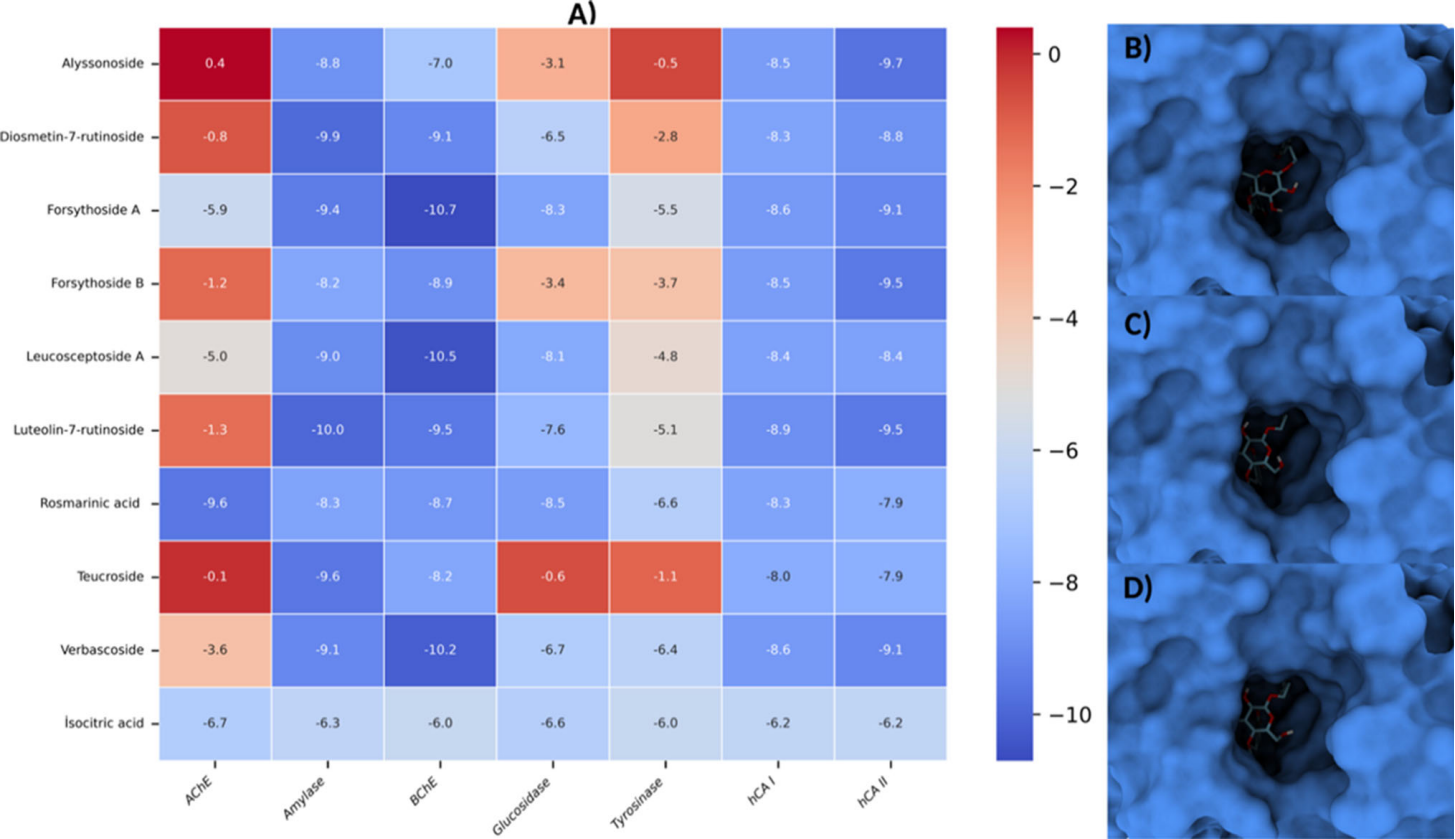
Docking profile of Teucrium‐derived phenylethanoid and flavonoid glycosides. (A) Heat map of binding energies (kcal/mol) for all ligand–enzyme complexes. (B) BChE–Forsythoside A. (C) BChE–Leucosceptoside A. (D) BChE–Verbascoside.

A library composed of the phenylethanoid and flavonoid glycosides forsythoside A, forsythoside B, leucosceptoside A, verbascoside, teucroside, alyssonoside, rosmarinic acid, luteolin‐7‐rutinoside, isocitric acid, and diosmetin‐7‐rutinoside, which were selected from *T. scordium* subsp. *scordioides*, was systematically screened against seven key therapeutic targets: BChE, AChE, *α*‐amylase, *α*‐glucosidase, tyrosinase and, hCA I, hCA II, using AutoDock Vina. Of the 70 ligand–enzyme complexes examined, 41 exhibited binding affinities of ≤−7.0  kcal/mol, meeting the predefined threshold for strong binding. The BChE–Forsythoside A complex exhibited the lowest binding score of −10.7  kcal/mol, while the weakest interactions within the selected set clustered around −7.0 kcal/mol. RMSD values ranged from 0.0834 to 11.1594 Å (Table 6) (Figure 2A).

**TABLE 6:** The docking score (kcal/mol) and interacting residues of the protein.

Compound	Target	PDB ID	Binding energy	RMSD	Type and binding site
Forsythoside A	BChE	6EQP	−10.7	0.6	Hbond: TRP:82; GLY:115; GLY:117; THR:120; THR:120; TYR:128; TYR:128; GLU:197; SER:198; TRP:430
					Hydrophobic: TRP:82; TRP:82; TRP:231; LEU:286; LEU:286; ALA:328; TYR:332; TRP:430
					Pi‐stacking: PHE:329
Leucosceptoside A	BChE	6EQP	−10.5	0.1	Hbond: TRP:82; GLY:115; GLY:115; GLY:117; THR:120; SER:198; SER:198; TRP:430; TYR:440
					Hydrophobic: TRP:82; TRP:82; LEU:286; ALA:328; PHE:329; TYR:332; PHE:398; TRP:430
					Pi‐stacking: TRP:231; TRP:231; PHE:329
Verbascoside	BChE	6EQP	−10.2	0.8	Hbond: ASP:70; GLY:78; TRP:82; GLY:117; THR:120; GLU:197; SER:198; TRP:430
					Hydrophobic: TRP:82; TRP:82; TRP:231; LEU:286; ALA:328; TYR:332; PHE:398; TRP:430
					Pi‐stacking: PHE:329
Luteolin‐7‐rutinoside	Amylase	2QV4	−10.0	5.9	Hbond: HIS:101; ALA:198; HIS:201; HIS:305; HIS:305
					Hydrophobic: TRP:59; THR:163; LEU:165
					Pi‐stacking: TRP:59; TRP:59
Diosmetin‐7‐rutinoside	Amylase	2QV4	−9.9	0.5	Hbond: ALA:106; GLY:164; ARG:195; HIS:299; ASP:300
					Hydrophobic: TRP:59; LEU:165; ILE:235
					Pi‐stacking: TRP:59
Alyssonoside	hCA II	4IWZ	−9.7	0.5	Hbond: HIS:4; TRP:5; ARG:58; ASN:62; ASN:67; GLU:69; GLN:92; HIS:94; HIS:96; HIS:119; THR:199; THR:200; THR:200; THR:200
					Hydrophobic: TRP:5; ASN:62; HIS:64; VAL:121; VAL:121; LEU:141; VAL:143; LEU:198
					Pi‐stacking: HIS:64; HIS:94
Rosmarinic A	AChE	7E3H	−9.6	0.3	Hbond: GLY:121; GLY:122; TYR:124; TYR:133; GLU:202; TYR:337
					Hydrophobic: TRP:86; TRP:86; TYR:124; PHE:297; PHE:297; PHE:338; PHE:338
Teucroside	Amylase	2QV4	−9.6	0.5	Hbond: TRP:59; GLN:63; ARG:195; ASP:197; ALA:198; LYS:200; GLU:233; GLU:233; ILE:235; HIS:305; HIS:305
					Hydrophobic: TRP:59; TYR:151; LEU:162; THR:163; LYS:200; ILE:235; ILE:235; ILE:235
					Pi‐stacking: HIS:201
					Salt bridge: LYS:200
Luteolin‐7‐rutinoside	BChE	6EQP	−9.5	0.4	Hbond: ASP:70; TRP:82; GLY:115; GLY:117; TYR:128; TYR:128; LEU:286; TYR:440
					Hydrophobic: TRP:82; TRP:430
Luteolin‐7‐rutinoside	hCA II	4IWZ	−9.5	0.9	Hbond: ASN:62; ASN:67; GLN:92; THR:199; THR:199; THR:199; THR:200; THR:200
					Hydrophobic: HIS:64; ALA:65; VAL:121; VAL:121; VAL:143; LEU:198; THR:200
					Pi‐stacking: HIS:94
					Salt bridge: HIS:64
Forsythoside B	hCA II	4IWZ	−9.5	0.5	Hbond: ASN:62; ASN:62; HIS:64; ASN:67; ASN:67; ASN:67; GLU:69; GLN:92; HIS:119; THR:199; THR:200; THR:200; THR:200; THR:200
					Hydrophobic: TRP:5; TRP:5; HIS:64; VAL:121; PHE:131; VAL:143; LEU:198; THR:200
					Pi‐stacking: HIS:94
Forsythoside A	Amylase	2QV4	−9.4	1.1	Hbond: TYR:62; GLN:63; ASP:197; ALA:198; SER:199; LYS:200; GLU:233; ASP:300; HIS:305
					Hydrophobic: TRP:59; TYR:62; LYS:200; LYS:200; ILE:235; ILE:235
					Pi‐stacking: HIS:201
					Salt bridge: HIS:305
Diosmetin‐7‐rutinoside	BChE	6EQP	−9.1	0.5	Hbond: ASP:70; ASP:70; TRP:82; SER:198; SER:287; ALA:328; TRP:430; TYR:440
					Hydrophobic: TRP:82; TRP:82; THR:120; PHE:329
Verbascoside	hCA II	4IWZ	−9.1	0.7	Hbond: ASN:62; ASN:67; ASP:72; ASP:72; GLN:92; HIS:119; THR:199; THR:200; THR:200
					Hydrophobic: GLU:69; ILE:91; ILE:91; VAL:121; VAL:143; LEU:198
					Pi‐stacking: HIS:94
Verbascoside	Amylase	2QV4	−9.1	0.6	Hbond: ALA:198; LYS:200; GLU:233; GLU:233; ILE:235; ASP:300; HIS:305
					Hydrophobic: TRP:59; TRP:59; TRP:59; LEU:162; LEU:162; LEU:165; LYS:200; ILE:235
					Pi‐stacking: HIS:201
					Salt bridge: HIS:305
Forsythoside A	hCA II	4IWZ	−9.1	0.9	Hbond: TYR:7; HIS:64; GLU:69; PHE:95; HIS:96; HIS:119; THR:199; THR:200; THR:200; THR:200; THR:200; ASN:244
					Hydrophobic: TRP:5; HIS:64; ALA:65; VAL:121; VAL:121; PHE:131; PHE:131; VAL:143; LEU:198; THR:200
					Pi‐stacking: HIS:94; HIS:94
					Salt bridge: HIS:64
Leucosceptoside A	Amylase	2QV4	−9.0	0.5	Hbond: GLN:63; THR:163; ALA:198; LYS:200; GLU:233; ASP:300; HIS:305
					Hydrophobic: TRP:59; TRP:59; TYR:62; ILE:235; ILE:235
					Pi‐stacking: TRP:59; TYR:151
					Salt bridge: HIS:305
Forsythoside B	BChE	6EQP	−8.9	3.3	Hbond: ASN:68; ASP:70; TRP:82; GLY:117; THR:120; THR:120; GLY:121; TRP:430; HIS:438
					Hydrophobic: TRP:82; TRP:231; LEU:286; PHE:398
					Pi‐stacking: PHE:329
Luteolin‐7‐rutinoside	HCA I	3LXE	−8.9	0.6	Hbond: HIS:64; ASN:69; GLN:92; HIS:94; HIS:96; HIS:119; THR:199; THR:199; THR:199
					Hydrophobic: LEU:198
					Pi‐stacking: HIS:67; PHE:91
					Salt bridge: HIS:94; HIS:200
Alyssonoside	Amylase	2QV4	−8.8	0.3	Hbond: GLN:63; TYR:151; ALA:198; SER:199; LYS:200; GLU:233; GLU:233; GLU:233
					Hydrophobic: TRP:59; TRP:59; TYR:62; LEU:162; LEU:165; LYS:200; LYS:200; ILE:235; ILE:235
					Pi‐stacking: HIS:201
					Salt bridge: HIS:305
Diosmetin‐7‐rutinoside	hCA II	4IWZ	−8.8	0.5	Hbond: TRP:5; ASN:62; HIS:64; ASN:67; ASN:67; ASN:67; GLN:92
					Hydrophobic: GLU:69; GLU:69; ILE:91
Rosmarinic A	BChE	6EQP	−8.7	0.2	Hbond: ASP:70; ASP:70; TRP:82; TRP:82; GLY:115; THR:120; TYR:128; TRP:430
					Hydrophobic: ASP:70; THR:120
					Pi‐stacking: TRP:82; TRP:82
Forsythoside A	HCA I	3LXE	−8.6	1.1	Hbond: HIS:64; GLN:92; HIS:94; HIS:96; HIS:119; THR:199; THR:199; THR:199; HIS:200; PRO:201
					Hydrophobic: VAL:62; VAL:62; PHE:91; VAL:143; VAL:143; LEU:198; TRP:209
					Pi‐stacking: HIS:94
					Salt bridge: HIS:67
Verbascoside	HCA I	3LXE	−8.6	0.9	Hbond: GLN:92; HIS:94; HIS:96; LYS:170
					Hydrophobic: VAL:62; ALA:135; LEU:141; LEU:198
					Pi‐stacking: PHE:91
					Salt bridge: HIS:64; HIS:67; HIS:200
Forsythoside B	HCA I	3LXE	−8.5	0.8	Hbond: HIS:64; SER:65; ASN:69; ASN:69; GLN:92; THR:199; THR:199; HIS:200; HIS:200
					Hydrophobic: LEU:198; HIS:200
					Pi‐stacking: HIS:94
					Salt bridge: HIS:64; HIS:67; HIS:200
Rosmarinic a	Glucosidase	7KBJ	−8.5	6.6	Hbond: GLN:247; ASP:251; LEU:252; GLY:253; GLY:254; LYS:283; TYR:291
					Hydrophobic: LEU:252; TYR:291; TRP:331; LEU:344
					Salt bridge: ARG:352
Alyssonoside	HCA I	3LXE	−8.5	1.7	Hbond: LYS:57; HIS:64; ASP:72; GLN:92; HIS:96; HIS:119; THR:199; THR:199; THR:199; THR:199
					Hydrophobic: PHE:91; PHE:91; LEU:131; LEU:198; PRO:202; TYR:204; TRP:209
					Pi‐stacking: HIS:94
					Salt bridge: HIS:64; HIS:67
Leucosceptoside A	HCA I	3LXE	−8.4	0.9	Hbond: GLN:92; HIS:94; HIS:96
					Hydrophobic: TRP:5; ALA:135; LEU:141
					Pi‐stacking: PHE:91
					Salt bridge: HIS:64; HIS:67; HIS:200
Leucosceptoside A	hCA II	4IWZ	−8.4	0.5	Hbond: ASN:67; GLN:92; HIS:96; HIS:119; THR:199; THR:200; THR:200
					Hydrophobic: ASN:62; VAL:121; VAL:121; VAL:143; LEU:198
					Pi‐stacking: HIS:94
					Salt bridge: HIS:64
Rosmarinic A	Amylase	2QV4	−8.3	0.7	Hbond: GLN:63; THR:163; ARG:195; HIS:299; ASP:300; ASP:300; ASP:300; HIS:305
					Hydrophobic: TRP:58; TRP:59; TYR:62; TYR:62; LEU:162; LEU:165
Diosmetin‐7‐rutinoside	HCA I	3LXE	−8.3	0.5	Hbond: ASN:61; HIS:64; GLN:92; GLN:92; HIS:94; HIS:96; GLY:171
					Hydrophobic: TRP:5; VAL:62; VAL:62; ALA:135
					Salt bridge: HIS:67
Rosmarinic A	HCA I	3LXE	−8.3	0.6	Hbond: SER:65; SER:65; PHE:66; PHE:95; HIS:119; THR:199; THR:199; THR:199; HIS:200
					Hydrophobic: PHE:91; ALA:121; VAL:143; LEU:198; HIS:200; TRP:209
					Pi‐stacking: HIS:64; HIS:94; HIS:94
Forsythoside A	Glucosidase	7KBJ	−8.3	9.1	Hbond: GLN:247; LEU:252; GLY:253; GLY:253; GLY:254; LYS:283; TYR:291; LEU:344; LEU:344; ALA:345; PHE:350
					Hydrophobic: LEU:252; ILE:327; PHE:330; TRP:331
					Salt bridge: ARG:334
Teucroside	BChE	6EQP	−8.2	nd	Hbond: ASP:70; TRP:82; GLY:115; GLY:116; GLY:116; THR:120; GLY:121; THR:122; TYR:128; LEU:286; HIS:438
					Hydrophobic: ASP:70; TRP:82; TRP:82; LEU:286; PHE:329; PHE:398; TRP:430
					Pi‐stacking: TRP:231; TRP:231
					Salt bridge: HIS:438
Forsythoside B	Amylase	2QV4	−8.2	4.0	Hbond: GLN:63; ARG:195; GLU:233; ASP:300; ASP:356
					Hydrophobic: TRP:58; TRP:59; TRP:59; TYR:62; LEU:162; ILE:235
					Salt bridge: HIS:305; HIS:305
Leucosceptoside A	Glucosidase	7KBJ	−8.1	nd	Hbond: TRP:236; GLY:240; GLY:264; ARG:267; ARG:267; PHE:271; LYS:283; TYR:348; ARG:352
					Hydrophobic: PHE:237; LEU:252; GLU:270; TYR:291
					Salt bridge: LYS:283; ARG:352
Teucroside	HCA I	3LXE	−8.0	5.2	Hbond: LYS:57; HIS:64; PHE:70; PHE:70; ASP:72; PHE:91; ARG:173; ARG:173; THR:199; PRO:201
					Hydrophobic: LEU:198; LEU:198; LEU:198
					Salt bridge: LYS:57; HIS:67; HIS:67; HIS:94; HIS:96; HIS:119
Teucroside	hCA II	4IWZ	−7.9	11.2	Hbond: TRP:5; ASN:67; ASN:67; PHE:70; PHE:70; ASP:72; ASP:72; GLN:92; ASP:130; PHE:131; GLY:132
					Hydrophobic: TRP:5
					Salt bridge: HIS:64
Rosmarinic A	hCA II	4IWZ	−7.9	1.1	Hbond: TYR:7; ASN:62; ASN:67; GLN:92; HIS:94; HIS:96; HIS:119; THR:199; THR:199; THR:200
					Hydrophobic: TRP:5; HIS:64; VAL:121; LEU:198
					Pi‐stacking: HIS:94
Luteolin‐7‐rutinoside	Glucosidase	7KBJ	−7.6	5.3	Hbond: GLU:248; ILE:250; LEU:252; GLY:253; GLY:264; ALA:345; TYR:348; TYR:348
					Hydrophobic: TYR:291; PHE:330; LEU:344; TYR:348; PHE:350
					Salt bridge: LYS:283; ARG:352; ARG:352
					Pi‐cation: ARG:334
Alyssonoside	BChE	6EQP	−7.0	6.4	Hbond: TRP:82; TRP:82; GLY:115; THR:122; TYR:332
					Hydrophobic: ASP:70; TRP:82; PHE:329; PHE:398
					Pi‐stacking: TRP:231; TRP:231; PHE:329
					Salt bridge: HIS:438

Among the complexes showing favorable docking scores, the BChE series exhibited the greatest abundance of strong binders, which were ranked as follows: Forsythoside A > Leucosceptoside A > Verbascoside  >  Luteolin‐7‐rutinoside  >  Diosmetin‐7‐rutinoside  >  Rosmarinic acid (Figure 2B–D). The active‐site residues Trp82, Gly115, Gly117, and Thr120 formed a dense network of hydrogen bonds and *π*–*π* stacking that stabilized the ligand near the catalytic triad, consistent with the known geometry of the BChE pocket [2, 3]. The rank order for amylase was as follows: Diosmetin‐7‐rutinoside  >  Teucroside  >  Forsythoside A > Verbascoside  >  Leucosceptoside A > Alyssonoside  >  Rosmarinic acid. Binding mainly involved the Ala106–Gly164–Arg195–His299–Asp300 region, where multiple hydrophilic bridges mimicked natural substrate interactions, indicating strong anchoring within the catalytic groove.

For AChE, only one complex met both thresholds: Rosmarinic acid, bridging the Gly121–Gly122–Tyr124–Tyr133–Glu202 residues that link the peripheral anionic site with the catalytic gorge. This arrangement matches previously described cholinesterase pocket topologies [51]. In the hCA I, Luteolin‐7‐rutinoside bound near His64–Asn69–Gln92–His94–His96–His119–Thr199, coordinating with the zinc ion through hydrophilic and aromatic contacts, while in hCA II, Alyssonoside ranked first, followed by Luteolin‐7‐rutinoside and Forsythoside B, each stabilized by hydrogen bonding around His4–Trp5–Arg58–Asn62–Asn67–Glu69 near the catalytic metal center [52, 53, 54]. In contrast, neither *α*‐glucosidase nor tyrosinase produced ligands that met both the energetic and geometric criteria. This is likely due to pocket steric constraints in intestinal *α*‐glucosidases and the binuclear type III copper requirements of tyrosinase. Accordingly, smaller *o*‐diphenolic/catecholic scaffolds are often better suited to the CuA/CuB site [55, 56, 57, 58, 59].

Overall, the docking analysis suggested that several phenylethanoid and flavonoid glycosides may interact favorably with selected enzyme targets, with BChE and *α*‐amylase showing comparatively stronger predicted binding patterns. These findings should be interpreted as computational hypotheses only, since docking scores do not constitute experimental proof of inhibition. Therefore, the selected complexes were used only as representative models for subsequent MD evaluation.

### 3.6 Molecular Dynamics Simulation

Five docked complexes achieving the highest scores were selected for in silico validation. As shown in Figure 1, the following substances were identified: Forsythoside A–BChE (C1), luteolin‐7‐rutinoside–amylase (C2), alyssonoside–hCA II (C3), rosmarinic acid–AChE (C4), and luteolin‐7‐rutinoside–hCA I (C5). Each system was propagated for 100 ns, during which the following parameters were monitored. The following parameters are to be considered: RMSD, root mean square fluctuation (RMSF), solvent accessible surface area (SASA), minimum distance, and hydrogen bond (Hbond) counts. MM/GBSA binding‐free energies were estimated for systems C1, C3, C4, and C5. In conclusion, the trajectories indicate stable protein scaffolds with complex‐specific ligand behaviors that are predictive rather than definitive.

**FIGURE 3:**
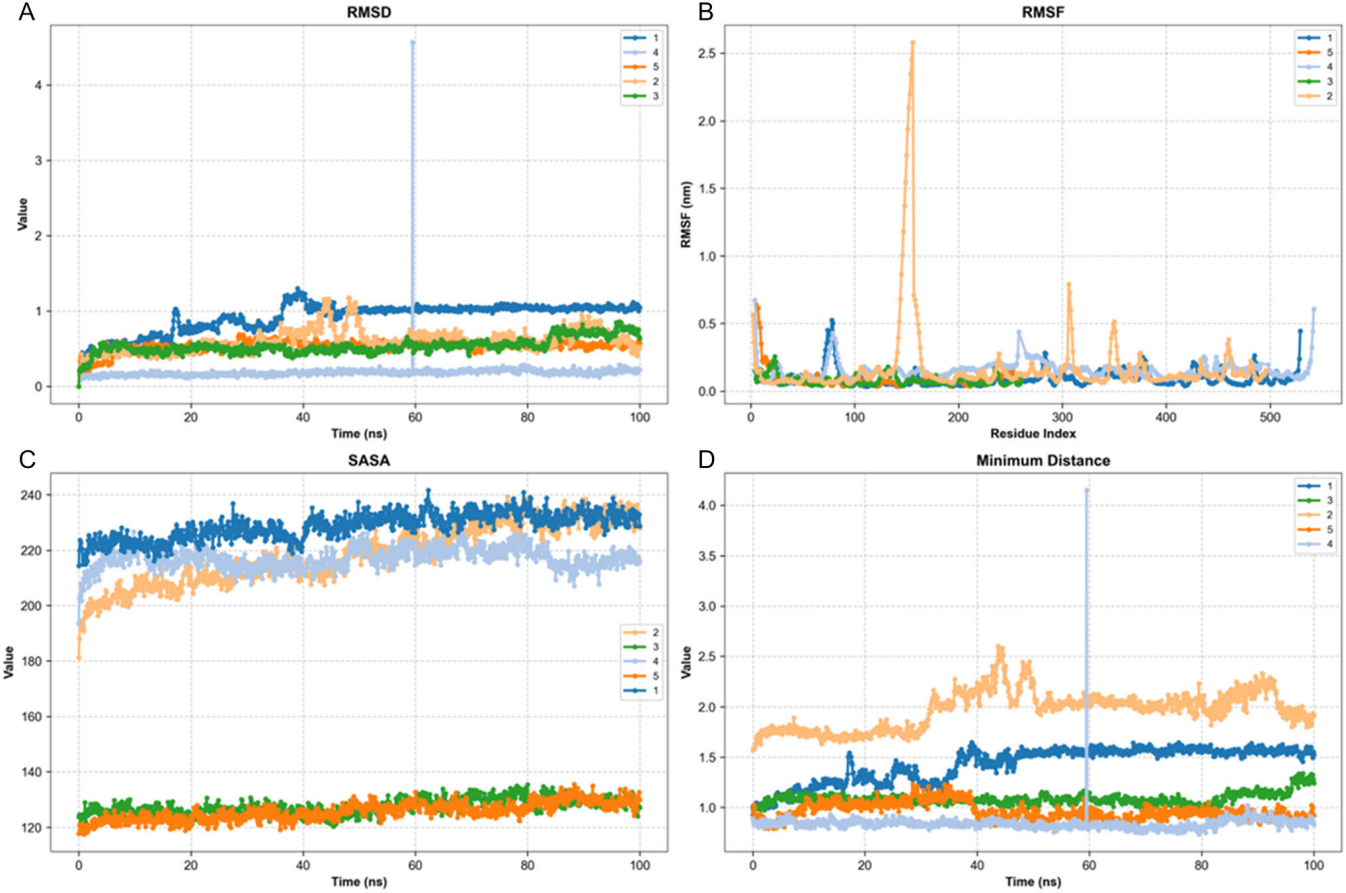
MD stability metrics for selected complexes: (A) RMSD, (B) RMSF, (C) SASA, (D) minimum distance. RMSD = Root mean square deviation; RMSF = root mean square fluctuation; SASA = solvent accessible surface area.

**FIGURE 4:**
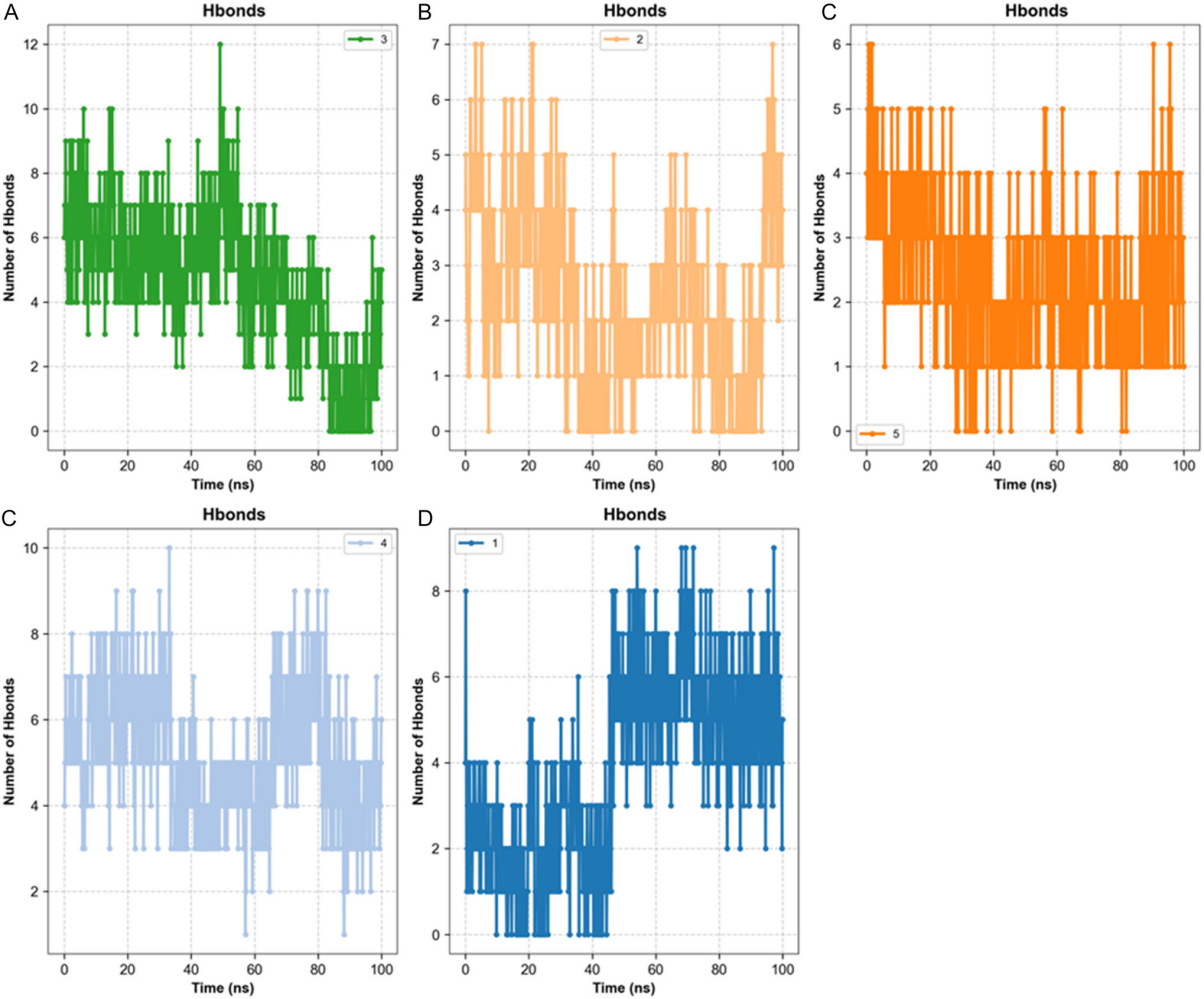
Time‐resolved protein–ligand hydrogen‐bond occupancy.

**FIGURE 5:**
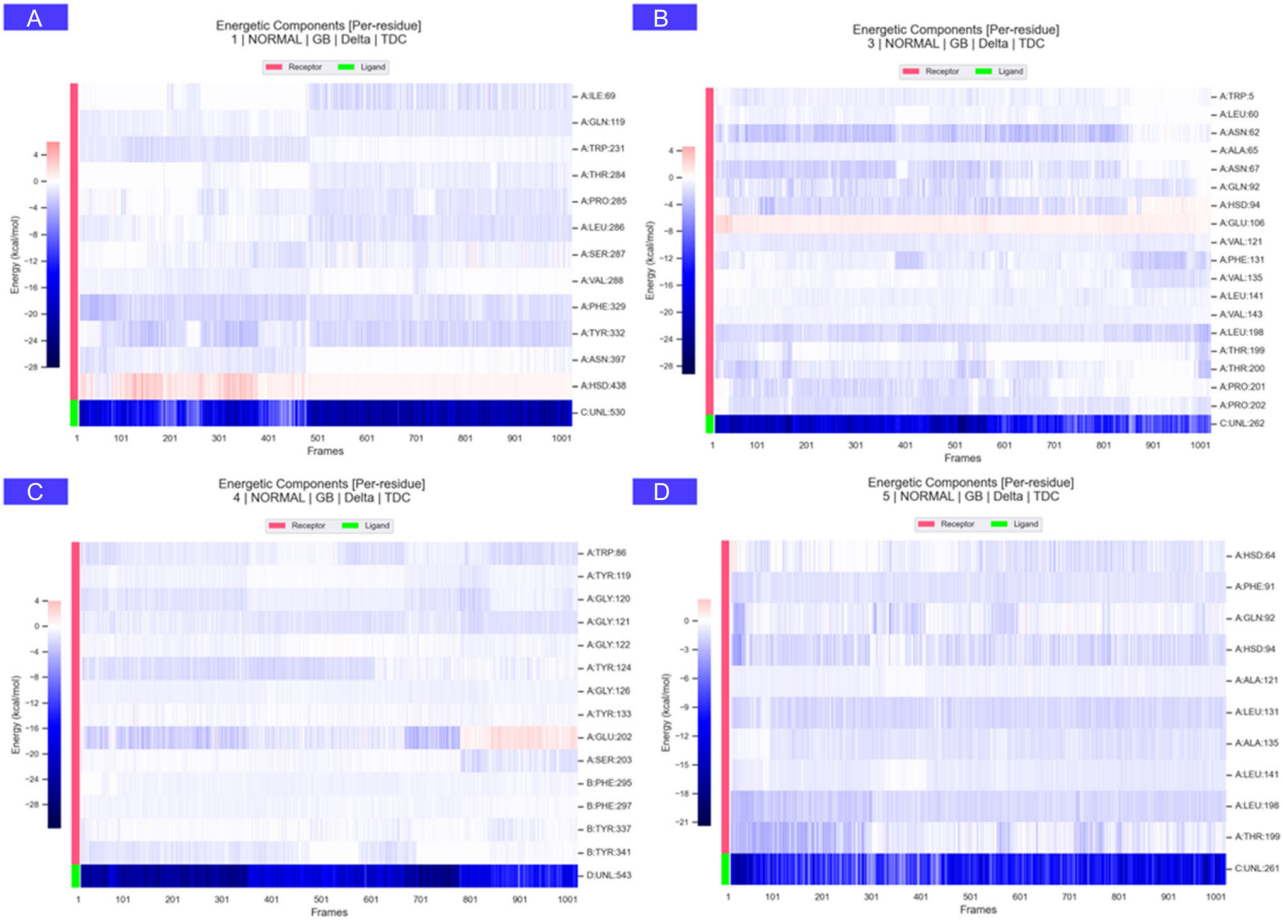
MM/GBSA binding‐free energies and per‐residue energy contributions.

C1 exhibited a low mean RMSD of ≈0.18 Å, modest RMSF values, a gradual increase in SASA from ≈195 to ≈220 nm^2^, a minimum distance of ≈0.84 Å, and sustained contact. The hydrogen bond exhibited remarkable strength and persistence, as shown in Figures 3 and 4. It is predicted that the interaction pattern will involve Ile69, Gln119, Trp231, Thr284, Pro285, Leu286, Ser287, Val288, Phe329, Tyr332, Asn397, and His438. The C3 model exhibited a RMSD of ≈0.53 Å, a low RMSF, and a SASA confined to the 120–130 nm^2^ range. It demonstrated a minimum distance of around 0.96 Å with uninterrupted contact and a steady yet low‐to‐moderate number of hydrogen bonds. The following amino acids have been predicted to form contacts: His94, Glu106, Val121, Phe131, Thr199, Trp5, Leu60, Asn62, Ala65, Asn67, Gln92, Val135, Leu141, Val143, Thr200, Pro201, Pro202, and Leu198. Despite a longer average minimum distance of ≈1.97 Å, C5 remained compact with an RMSD of ≈0.53–0.61 Å, low RMSF, a SASA of ≈127 nm^2^, and continuous contact. Hbonds were found to be modest but consistent, and residues predicted to contribute include His64, Phe91, Gln92, His94, Ala121, Leu131, Ala135, Leu141, Leu198, and Thr199. C4 demonstrated intermediate stability, evidenced by an RMSD of ≈0.60 Å, a low‐to‐moderate RMSF, and an increase in SASA from ≈219–229 nm^2^. The minimum distance was ≈1.43 Å, with sustained contact, and the Hbond count was moderate. The predicted contacts included Trp86, Tyr119, Tyr124, Tyr133, Glu202, Ser203, Phe295, Phe297, Tyr337, Tyr341, Gly121, and Gly122. C2 exhibited a weaker dynamic profile. Terminal RMSD approached ≈0.95 Å, RMSF peaked at ≈2.58 Å around residues 152–156, SASA increased from ≈194–205 to ≈220 nm^2^, and the Hbond count decreased over time while the minimum distance remained short (≈1.09 Å). Due to the absence of these less mature dynamics, MM/GBSA was not performed for C2 (see Figure 5).

The MD outcomes were grouped into three performance categories based on computed metrics. The group with the highest performance included C1, C3, and C5. C1 exhibited a distinctly stable profile, characterized by low RMSD and RMSF values, a short and continuous minimum distance, and a robust, persistent hydrogen‐bond network accompanied by a balanced increase in SASA. The predicted binding residues Trp231, Phe329, Ser287, Asn397, and His438 appeared to stabilize the ligand within the active‐site cavity of BChE. It was observed that both C3 and C5 maintained compact conformations with low RMSD and stable SASA profiles, while preserving uninterrupted minimum distance trajectories. The hydrogen‐bond networks exhibited low‐to‐moderate intensity, yet were consistently maintained. In C3, the predominant predicted interactions were with His94, Glu106, and Thr199, whereas in C5, the key contacts likely involved His64, His94, and Thr199, indicating stable coordination in the vicinity of the zinc‐binding pocket. The intermediate group was represented by C4. RMSD remained within a moderate range, and although SASA showed a slight increase, the minimum distance persisted at short values, and the hydrogen‐bond network was sufficient to preserve the ligand pose. The primary interacting residues were Trp86, Tyr119, Tyr124, Tyr133, Glu202, Ser203, Phe295, Phe297, Tyr337, and Tyr341, suggesting stable yet solvent‐exposed binding. The group with the lowest performance was C2. The findings indicated elevated loop flexibility, declining hydrogen‐bond occupancy, and an increasing SASA trend, suggesting partial instability in the bound state. Despite the minimum distance remaining minimal, the elevated structural noise impeded adequate equilibration, consequently hindering reliable energy estimation. The in silico findings indicate that, while C1, C3, and C5 display stable and compact binding behavior, C4 exhibits moderate stability, and C2 requires further optimization to enhance conformational consistency and solvent adaptability before energetic validation (Figures 3, 4, and 5).

The MM/GBSA ranking was generally in agreement with the trends observed for RMSD, RMSF, SASA, minimum distance, and hydrogen‐bond profiles, with C1 showing the most favorable overall computational behavior among the analyzed systems. Nevertheless, these results should be interpreted cautiously, as MD and MM/GBSA provide supportive computational evidence rather than definitive confirmation of inhibitory activity. Taken together, the docking and simulation data suggest that some of the analyzed glycosides may maintain stable binding modes under dynamic conditions and may warrant future target‐specific experimental testing using isolated compounds. For C3 and C5, it is hypothesized that fine‐tuning of the aglycone/sugar ratio without disrupting the metal coordination pharmacophore will enhance interaction efficiency. In the case of C4, the introduction of small aromatic derivatives capable of reinforcing *π*–*π* stacking interactions may result in a strengthening of binding affinity. Conversely, C2 would benefit from the design of more compact, loop‐compatible analogs with donor–acceptor arrangements that enhance water‐mediated stabilization. Once RMSD and Hbond stability are improved, MM/GBSA reassessment would become more meaningful. The findings of this study indicate that the binding architectures predicted during the docking stage are largely retained under dynamic conditions and furthermore highlight C1 as the most promising scaffold for the rational design of multitarget enzyme inhibitors.

## 4 Conclusion

To our best knowledge, this is the first report on the antioxidant and enzyme inhibitory properties of *T. scordium* subsp. *scordioides*. Comprehensive chemical analysis revealed that the plant was rich in phenolics. Phenylethanoid glycosides, mainly detected in polar extracts, were the major constituent of the species followed, respectively, by flavonoid glycosides and phenolic acids. The species exerted potent antioxidant activity with the three polar extracts had significantly higher effect than the EtOAc extract in the majority of assays. The enzyme inhibitory property varied according to the tested enzyme and extract, indicating that the polarity of solvents significantly affects the extraction effectiveness and must be taken into account for their biological properties. These findings showed that *T. scordium* subsp. *scordioides* could be a promising source of active ingredients’ for different pharmaceutical and cosmetic applications. Furthermore, the integrated docking, MD, and MM/GBSA analyses provided a preliminary computational framework for understanding the possible interactions of the major glycosides with multiple enzyme targets. These in silico findings should be considered supportive and hypothesis generating, and they require confirmation through bioassays performed with isolated compounds and target‐specific validation studies. In vivo studies and isolation of bioactive compounds as well as determination of their mechanism of action are recommended.

## Supporting Information

Additional supporting information can be found online in the Supporting Information section.

## Funding

This work was supported by the University of Jaén (UJA) (PID2024‐160229OB‐I00 and R1B_2025_024).

## Conflicts of Interest

The authors declare no conflicts of interest.

## Declaration of Generative AI and AI‐Assisted Technologies in the Writing Process

In the preparation of this manuscript, the authors utilized AI‐based language tools (Grammarly and ChatGPT‐4) for initial language editing and proofreading. The authors subsequently reviewed and refined all content, and they assume full responsibility for the final work and its conclusions.

## Supporting information

Supplementary Material

## Data Availability

Most of the data generated or analyzed during this study are included in this published article. Specific datasets for chromatographic analyses are available from E.J. Llorent‐Martínez (email: ellorent@ujaen.es) on reasonable request.
