# Supplementary material for: Chemical Constituents and Biological Potentials of Teucrium scordium subsp. scordioides Extracts: In Vitro Experimental and In Silico Perspectives
Source: ChemistryOpen. 2026 Apr 28;15(5):e70216. doi: 10.1002/open.70216 (PMC13125859; doi:10.1002/open.70216)
Supplement: Supplementary file 1 — Supplementary Material [file OPEN-15-e70216-s001.pdf]

Phytochemical analysis by HPLC-ESI-Q-TOF-MS

Approximately 5 mg of each dried extract was dissolved in 1 mL of methanol (except the aqueous extract, which was re-dissolved in 1 mL of 10% MeOH) and filtered through 0.45 μm membrane filters. An Agilent 1200 (Agilent Technologies, Santa Clara, CA, USA) equipped with an Agilent 6530B Q-TOF-MS was used for the characterization. The analyses were performed using a Luna Omega Polar C_{18} column (150 × 3.0 mm; 5 μm particle size) with a Polar C18 Security Guard cartridge of 4 × 3.0 mm (Phenomenex, Torrance, CA, USA). The HPLC gradient elution program was carried out with mobile phases of water + formic acid 0.1% *v*/*v* (eluent A) and acetonitrile (eluent B). The gradient program was as follows: 10-25% B in 0-25 min, 25% B in 25-30 min, 25-100% B in 30-35 min, and 100% B (35-37 min); then, eluent B was returned to 10% with a 7 min stabilization time. The flow rate was 0.4 mL min^{-1}, an injection volume of 10 μL was used, and analyses were performed at room temperature. The parameters used were capillary voltage, 3500 V; nebulizer pressure of 45 psi; drying gas flow rate, 10 L/min; gas temperature, 325 °C; skimmer voltage, 60 V; and fragmentor voltage, 140 V. Data were acquired in negative ion mode using an orthogonal ESI source. The MS and auto MS/MS modes (collision energies of 10, 20, and 40 V) were set to acquire *m*/*z* values between 50 and 1200 at a scan rate of 2 and 3 spectra per second, respectively. Agilent Mass Hunter Qualitative Analysis software version B.06.00 was used for post-acquisition data processing. Molecular formulas, errors (ppm), and fragment ions were obtained by the tool Generate Formulas using the Auto MS/MS algorithm.

For the quantitative discussion, Extracted Ion Chromatograms (EICs) in MS mode were obtained for each compound at its deprotonated molecular ion (using a symmetric expansion of ±5 ppm), and integrated peak areas were used to compare phytochemical profiles between leaves, as well as the extraction efficiency of the different solvents. Relative standard deviations were lower than 5% in all cases.
